# The Adaptation of a Youth Diabetes Prevention Program for Aboriginal Children in Central Australia: Community Perspectives

**DOI:** 10.3390/ijerph18179173

**Published:** 2021-08-31

**Authors:** Athira Rohit, Leisa McCarthy, Shiree Mack, Bronwyn Silver, Sabella Turner, Louise A. Baur, Karla Canuto, John Boffa, Dana Dabelea, Katherine A. Sauder, Louise Maple-Brown, Renae Kirkham

**Affiliations:** 1 Menzies School of Health Research, Charles Darwin University, Casuarina 0810, Australia; athira.rohit@menzies.edu.au (A.R.); leisa.mccarthy@menzies.edu.au (L.M.); shiree.mack@menzies.edu.au (S.M.); 2 Aboriginal Community-Controlled Health Organization, Central Australian Aboriginal Congress, Alice Springs 0870, Australia; bronwyn.silver@caac.org.au (B.S.); sabella.turner@caac.org.au (S.T.); john.boffa@caac.org.au (J.B.); 3 Child & Adolescent Health, Sydney Medical School, University of Sydney, Sydney 2006, Australia; louise.baur@health.nsw.gov.au; 4 Wardliparingga Aboriginal Health Equity, South Australian Health and Medical Research Institute, Adelaide 5001, Australia; Karla.Canuto@sahmri.com; 5 Lifecourse Epidemiology of Adiposity and Diabetes (LEAD) Centre, University of Colorado Anschutz Medical Campus, Aurora, CO 80045, USA; DANA.DABELEA@CUANSCHUTZ.EDU (D.D.); katherine.sauder@cuanschutz.edu (K.A.S.); 6 Department of Endocrinology, Royal Darwin Hospital, Darwin 0810, Australia

**Keywords:** indigenous health and wellbeing, community consultation, prevention

## Abstract

This study reports on integrating community perspectives to adapt a family-focused, culturally appropriate behavioural intervention program to prevent diabetes among Aboriginal children (6–11 years) in Central Australia. A participatory action research approach was used to engage a range of service providers, cultural advisors, and family groups. Appropriateness, acceptability, content, and delivery of a prevention program within the Central Australian context were discussed through a series of workshops with twenty-five service providers and seven family groups separately. The data obtained were deductively coded for thematic analysis. Main findings included: (i) the strong need for a diabetes prevention program that is community owned, (ii) a flexible and culturally appropriate program delivered by upskilling community members as program facilitators, and (iii) consideration of social and environmental factors when implementing the program. It is recommended that a trial of the adapted prevention program for effectiveness and implementation is led by an Aboriginal community-controlled health service.

## 1. Introduction

T2DM and obesity in children and adolescents are escalating global concerns [1]. Obesity is a key risk factor for T2DM, with other contributing factors observed including intergenerational factors (maternal overweight/obesity and in utero exposure to hyperglycaemia) as well as social determinants of health [2,3,4,5]. In Australia, the incidence rates of T2DM are 18 times higher among Aboriginal and Torres Strait Islander children and adolescents compared to non-Aboriginal youth [6,7,8]. The recent national Aboriginal and Torres Strait Islander Health Survey reports a significant rise in the proportion of children aged 2–14 years old affected by overweight or obesity from 30% in 2012–2013 [9] to 37% in 2018–2019 [10]. Along with the identified risk factors, these high differences in the incidence rates of T2DM in Aboriginal and Torres Strait Islander youth need to be placed within the context of continuing intergenerational impacts of colonization [11,12] and the existing social determinants of health such as lack of access to affordable food choices and food insecurity experienced by the Aboriginal and Torres Strait Islander people living in remote communities [13,14,15]. Over centuries, the Aboriginal and Torres Strait Islander people in Australia have shown incredible strengths and resilience through their strong connections to language, culture and land [16]. Colonization has significantly impacted these connections experienced by Aboriginal and Torres Strait Islander people through loss of land, language and culture; significant poverty; intergenerational trauma; and a heavy burden of chronic conditions including T2D and obesity [11,12].

Prevention is critical to breaking the intergenerational cycle of metabolic risk and early intervention is crucial for effective outcomes [7]. The Lancet commission for adolescent health and wellbeing [17] recommends substantial investments in health care and prevention approaches to tackle the escalating incidence of youth-onset chronic conditions including obesity and T2DM. Engaging communities in the design of such prevention programs is equally critical [7,16,18]. Incorporation of independent community consultations ensures the program design is aligned with respective community priorities [16,18].

International evidence suggests that youth obesity rates track from 6–7 years of age into adulthood [19,20,21]. The key benefits of targeting this age group include greater potential for changing health behaviours before they are well-established and before metabolic changes of puberty [19,20,21]. Obesity prevention programs among First Nations children have had limited success globally [22,23,24] due to several factors including difficulty retaining program participants [24], lack of culturally appropriate venues and resources [22], and limited opportunities for partnership with communities [24]. Several school-based obesity preventions and/or management programs among American and Canadian First Nations communities have reported no change in body mass index or related risk factors among primary school aged children [22,23]. Lack of intense or sustained intervention programs was listed as a barrier in reporting zero change in body mass index (BMI). A novel home-based intervention for New Zealand children and adolescents aged 5–16 years, including 47% Māori participants, could not maintain sustained participation in the multidisciplinary intervention program (15% compared to 57% among New Zealand European participants) [24]. The program included a series of information sessions delivered by a physical activity coordinator, dietitian, and psychologist. To maintain sustained participation of Māori participants, the New Zealand study recommended Māori world views be incorporated into future interventions, with practical suggestions including use of culturally appropriate venues, and appropriate community consultations [24]. Similarly, in Australia, adaptations of obesity prevention programs for Aboriginal children have highlighted strength-based approaches including the need for culturally appropriate program content and delivery mode [25], to establish genuine partnerships with participating communities, [26], accommodate Aboriginal world views, and suitable evaluation frameworks prior to program implementation [27]. 

Clearly, there is a need for relevant and culturally appropriate prevention programs in Australia. The Tribal Turning Point (TTP), a behavioural intervention program, incorporated those strength-based characteristics and had been successfully implemented as a pilot program with American First Nations youth aged 7–10 years with overweight/obesity. It was adapted from the national Diabetes Prevention Program in the United States [28,29]. Key strategies of this program for this population group included (i) strong partnerships with communities, (ii) family-focused intervention (active participation of at least one caregiver and inclusion of siblings), (iii) age and culturally appropriate adaptation of the Diabetes Prevention Program [29], and (iv) high acceptability among participating families [21]. Other key characteristics of the TTP program are summarized in Table 1. The pilot study reported a significant reduction in body mass index z-score, with 84% intervention attendance and 97% retention over 8 months [21].

In 2018, the Diabetes across the Lifecourse Northern Australia Partnership’s (Partnership) Aboriginal and Torres Strait Islander Advisory Group set the importance of community-based initiatives to prevent youth-onset obesity and diabetes as a priority [30]. This priority was set in order to address the issue of increasing rates of youth-onset obesity and diabetes among the Aboriginal children. The current study was initiated in response to the priority and aimed to adapt the TTP program to develop a family-focused, culturally appropriate behavioural intervention program relevant for Aboriginal children in Central Australia. Key strengths of this proposed program include established, strong relationships between researchers, practitioners and communities in Central Australia (key partners have been working together for over fifteen years) and community leadership by Central Australian Aboriginal Congress (Congress), one of the most experienced Aboriginal primary health care services and a national leader in improving health outcomes for all Aboriginal people [31].

## 2. Materials and Methods

### 2.1. Reflexivity

Authors A.R. (PhD in Health Sciences), S.M. (Certificate II in community-based research) and R.K. (PhD in Public Health) conducted interviews and focus groups with a range of service providers and family groups in an urban setting in Central Australia. S.M. is a local Aboriginal woman, with experience in community consultation and Aboriginal social and emotional wellbeing. Being a local community member, S.M. was passionate about the proposed diabetes prevention program and her involvement was instrumental in providing a safe space for families and other community members to share their views and stories. R.K. is a non-Aboriginal social scientist with ten years’ experience in applying qualitative research methods in Aboriginal and Torres Strait Islander health research. A.R. is a non-Aboriginal researcher who managed this project under the supervision of R.K. A.R. was raised with an Indian cultural background, has expertise in conducting interviews in remote community settings and has five years’ experience in managing research projects.

### 2.2. Study Setting

Central Australia covers 65% of the total Northern Territory (NT) of Australia. The estimated resident population in Central Australia as of 2019 was 38,728, and 35% of the population was Aboriginal and Torres Strait Islander peoples [32,33]. Almost 60% of the Central Australian population resides in a major township called Alice Springs where the current community consultations were conducted. 

### 2.3. Study Design

A qualitative participatory action research methodology [34] was employed to obtain in-depth insight from key stakeholders and families to inform the program adaptation. A quality appraisal tool named CREATE [35] was used as a framework to report key elements of the current study through an Aboriginal and Torres Strait Islander cultural lens (please see Table 2). 

Key stakeholders were identified through the Partnership and included: health partners in Central Australia, including the Central Australian Aboriginal Congress (Congress), an Aboriginal Community Controlled Health Service, non-health Aboriginal community-controlled organizations, non-government and government organizations. Aboriginal families with children aged 6–11 years living in Alice Springs were identified through Congress in Central Australia and a local community health researcher (S.M.). S.M. and R.K. conducted preliminary consultations with family groups and service providers who participated in the study.

Ethics approval was obtained by the Central Australian Human Research Ethics Committee (CA-19-3343). Other approvals from Congress, Central Australian Health Services, other Primary Health Care services and the Northern Territory Department of Education were obtained prior to commencing the project. Participants were approached by S.M. or A.R. either by email, telephone, or face-to-face contact. Interviews or workshops with stakeholder participants occurred at participants’ workplaces and focus groups with Aboriginal families and Elders occurred in an open public space (community centre, park, etc.). All participants were provided with a participant information sheet and a verbal explanation regarding the project. With verbal and written consent from each participant, all interviews or workshops were audio recorded.

Data collection occurred in three stages. Stage one involved engaging service partners and families to assess the suitability of the intervention including program content and structure. Six two-hour workshops and two one-hour workshops were conducted among service providers and families, respectively. Stage two established working groups that involved representatives from the community, service providers and researchers. A technical reference group was formed to oversee the community consultations, revision of the program content and provide governance for implementation and monitoring of the revised and adapted TTP program. A dietitian working group was formed for nutrition- and diabetes-related content expertise. A cultural reference group was formed to oversee the cultural aspects and language used in the program. Terms of Reference for the technical reference group, dietitians working group and cultural reference group were developed where regular meeting times and duration were agreed by all members. Stage three focused on the practical supports, such as staff training, required to trial the intervention. Five workshops were undertaken with service providers and families separately to explore revised content, delivery mode, training modules for health coaches and realistic time frames. 

### 2.4. Data Analysis 

All researchers undertook data analysis and interpretation using an Aboriginal cultural lens to ensure that Aboriginal voices were prioritised [36]. Audio recordings from the consultation workshops were transcribed by an external provider and uploaded into NVivo (Version 11) software for data management and coding. A.R. completed the initial coding, which was cross-checked by S.M. and R.K. To further ensure transparency and accuracy of data interpretation, results from preliminary coding were presented back to service providers and families. Following this, a round of in-depth deductive coding was executed by A.R. and common themes identified.

## 3. Results

Eleven workshops with twenty-five service providers and three workshops with seven family groups were conducted (*n* = 38) across three stages of consultation. Characteristics of study participants are included in Table 3. The average time duration of workshops and focus groups was one hour. Major themes that emerged from the deductive coding of the consultation transcripts are outlined and compared with the key characteristics (Table 1) of the original TTP program below.

### 3.1. A Community Owned Prevention Program

From our consultations with community members and service providers, a diabetes prevention program targeting children and early adolescents was identified as an important priority in Central Australia. As described by an Aboriginal Elder, “This type of program would be a good idea for Central Australia.”

The preventive nature of the program was identified as a key priority:

“… it is very important for our children. We want them to be happy, to live happy, healthy lives. This [program] is very important because we got to try to hammer down our disease. It can be prevented early”—Aboriginal participant, Cultural advisor.

All participants unanimously suggested that an adapted program needs true representation from the local community. The importance of “getting elders from each community on board” with the program and the significance of “Congress [a local community-controlled organization] guiding the program” was highlighted. It was also recommended that the program needs to be owned and delivered by the local community members. As described by a parent, “I think people would engage more if they related to the person. So, definitely a local person”—Aboriginal participant, Parent. The significance of program evaluation was highlighted by the cultural advisors and indicated that “the program needs to be very strong, and it needs to make sure that is reflected and there is a review process” … “the review needs to be a very methodical, not rushed”. 

### 3.2. Local Facilitators Trained for Each Community

Participants suggested that local people from the community, such as Aboriginal Health Practitioners, need to be upskilled for delivering the program along with ongoing support from multiple health care professionals such as dietitians and diabetes educators. As iterated by an Aboriginal Health Promotion Officer, “you need that facilitator to be trained in various things, you know, the nutrition area and then you also get them cross-culturally trained that they’d be able to deliver culturally appropriately”. 

Participants emphasized that training is important for local facilitators to build their skills and capacity in delivering the program. In addition, as quoted by a participating Aboriginal cultural advisor, facilitators need to have “previous facilitation skills and be… able to go in workshops, being able to be very organized, be able to plan, have planning skills, and good ability to public speaking”.

Participants also highlighted several considerations to enhance the sustainability of the program. These included male and female facilitators, appropriate remuneration of facilitators and that they are represented as champions of the community to promote engagement. Aboriginal Health Practitioners, Aboriginal Liaison Officers, Aboriginal Education Officers and outreach health workers were suggested as being appropriate facilitators. Another suggestion by a non-Aboriginal Dietitian participant was to “get young [facilitators … like that big brother sort of approach, as in you’re more likely to get engaged when [buddying] young children with young adolescents.”

It was also suggested that a program manager would be required to provide centralised support for facilitators:

“I really think [facilitators], need to be well supported. If they’re senior staff members… they should be able to work independently obviously with some autonomy, but also have the backing and the guidance of a senior person”—Aboriginal participant, Cultural advisor.

### 3.3. Incentives Are Good for Positive Engagement

An incentive system for program participants was suggested by both service providers and community members as important for promoting ongoing encouragement for attendance, participation, and weekly goal attainment. A recommendation was that incentive items could promote healthy eating and physical activity, such as gift cards for sporting centres. As suggested by one Aboriginal participant (Parent), “The sports equipment’s a good one.”

### 3.4. A Flexible and Culturally Appropriate Program

Engaging the whole family was noted as a major strength of the program by family participants. An Aboriginal parent participant recommended that “the program needs to be flexible and dynamic” to enable any carer of the family to attend the program along with the child. Practical recommendations “about having something there [in the program] to assist the carers, parents, nannas” were highlighted by another Aboriginal parent participant. A common recommendation was to provide transport for families to and from the program location and was highlighted by an Aboriginal Elder participant that “this [transport] is the best way to support them [carers]”.

Another important consideration for the program is being flexible, as families have commitments and competing priorities that may impede their ability to attend the program regularly. This was well reflected by the Aboriginal Cultural advisor participant, “… we have a lot of interactions, we have sorry business (Rituals observed during a period of mourning by the Aboriginal and Torres Strait Islander peoples. This time involves responsibilities and obligations to attend funerals and participate in other cultural events, activities or ceremonies with the community. An Aboriginal and Torres Strait Islander community may also conduct Sorry Business if a family or community member is ill or imprisoned, or to mourn the loss of cultural connection to the land (e.g., if a native title application is lost), which can be as painful as the loss of a loved one. https://www.supportingcarers.snaicc.org.au/connecting-to-culture/sorry-business/ (accessed on 21 May 2021)…we have all these things to go to, like ceremonial leaves and we have women’s lore meeting and men’s business.” 

Several suggestions were raised by the participants to address long term attendance and continuous commitment of families to the whole program. Kinship (The kinship system is a system of Aboriginal social organisation and family relationships across Central Australia. The kinship system determines how people relate to each other and their roles, responsibilities and obligations in relation to one another, ceremonial business and land. The kinship system determines who marries who, ceremonial relationships, funeral roles and behaviour patterns with other kin https://www.clc.org.au/articles/info/aboriginal-kinship (accessed on 21 May 2021) structures influence attendance at programs, and as such, should be a consideration when planning and facilitating the program. It was suggested that small groups of participants (2–3 families) might be appropriate to promote program participation and that the best approach for recruitment is to leave it to the comfort and choice of the families. As iterated by an Aboriginal Health Promotion Officer, “We’ve got to be wary of the kinship, it is very strong in Central Australia. It is better to work individually with the families or … get all the families as different groups of families together”. Other strategies to enhance youth engagement included setting up youth groups and fun activities outside the program sessions for children attending the program. Participants also suggested breaking down program sessions and/or prioritizing content and delivering as a smaller program. It was also suggested the program be aligned with respective community sports weekends or other youth activities.

There was consensus among participants that weekly program sessions are important to encourage momentum and interest in the program. Several participants suggested primary schools to be an appropriate place for program delivery. Others questioned the inclusiveness or appropriateness of this environment, with concerns that it may not be a place where all parents feel comfortable enough to participate. A few participants suggested that as health clinics work closely with the family, they should deliver the program along with other services. The majority of families and a few health service providers recommended the program be delivered in a place that encourages family participation (such as town camps (There are 43 town camps across the Northern Territory of Australia. They are primarily areas set aside where Aboriginal people live in and around towns and cities. In some areas, the town camps were established in the 1970s and 1980s to provide a place where Aboriginal people visiting from remote areas could stay for short periods. Over time, some people have made the town camp their permanent home and have lived there for over a decade. https://tfhc.nt.gov.au/housing-and-homelessness/town-camps-and-community-living-areas/town-camps-review (accessed on 15 June 2021))). In line with this, a non-Aboriginal participant (Dietician) iterated that:

“… place-based delivery is probably going to be more effective, for example, at a town camp community centre and you had enough participants that were there, or enough people that potentially could participate, you probably will get the family groups if they’re around.”

It was suggested that the program could be delivered at the end of a school day aligning with the time for lunch or afternoon tea. The language and the content used need to be clear and interpreter services should be used throughout the program as required. Other recommendations included (i) to extensively use visual tools to engage families and children, (ii) to have direct focus on families’ priorities, and (iii) to use localized information of the specific community while preparing program resources.

It was also suggested that the program delivery needs to be more hands on rather than continuous lecturing (a few examples included bush walks and cooking sessions) and that talking about various study topics in small groups is preferred to large groups. As suggested by the Aboriginal Cultural advisor participant, “Have that good communication …. They’ll sit down and listen, but you might just listen to them first.” Another Aboriginal Community Engagement Officer participant suggested the program delivery to be “culturally appropriate for the communities... something very simple, simple terms, nothing complex. Be flexible, it must be flexible if you’re working with a family.” 

It was recommended the Aboriginal Health Promotion officers incorporate strategies for culturally appropriate program delivery that may include “hands-on stuff rather than sitting down and talking” and “having visual videos of young families or, you know, like Indigenous family videos… like this is what we’re aiming to do, and show that, this is what this family is done etc.”

### 3.5. Recommendations for Program Layout and Sessions

All participants agreed that the frequency (one session per week) and duration (2 h) of the original TTP program would be appropriate in the urban Central Australian context. Inviting Aboriginal people who had experienced chronic conditions, “like those ladies in renal, you know, the elders there, they can talk about their life experience around having that chronic condition” was suggested by the Aboriginal Cultural advisor participant and a few other participants.

Service providers and community members suggested individual family sessions as platforms for individual ‘yarning sessions’ and focused conversations for that particular family situation. This approach would assist in building trust and rapport between family members and program facilitators that is required for meaningful engagement. Booster sessions included in the TTP program were considered a good way of following up program participants however it was recommended that in Central Australia, the content included in booster sessions be more targeted according to the families’ needs.

All participants agreed that it is important to set simple and short-term goals for children and family members attending the program. For example, an Aboriginal parent recommended that “the participants can come up with their own goals to the program and ask [for] advice on how to achieve those goals”. Several suggestions to follow-up ‘family goals’ were suggested including using phone applications or closed social media groups or using simple surveys with tick boxes or smiley characters. Participants including health service providers, family groups and cultural advisors suggested that depending on the family circumstances, the process of ‘following-up’ could be time-consuming as iterated by an Aboriginal parent, it is important to “be more frequent in touch basing from facilitator’s side, phone calls on how things are going, etc. [with families].”

### 3.6. Addressing Social Determinants

Participants highlighted the importance of acknowledging existing social determinants of health in the community to promote successful implementation and meaningful outcomes of the adapted program. Affordability and easy access of healthy food and drinks was a key concern in the community. It was suggested by an Elder that “all shops need to be involved”. As iterated by a non-Aboriginal Dietitian participant, it is crucial to be “working with the settings—with the stores, with the hostels, the food supply” to enhance uptake of behaviours promoted by the adapted program.

## 4. Discussion

This consultation project informed the adaptation of the TTP program for Aboriginal children and their families in Central Australia. Our consultations indicated a strong need for such a program that is owned and led by the community. Other major findings included the need for trained local facilitators, culturally appropriate program delivery and acknowledgment of social determinants of health within the community while implementing the program.

The principle finding of our study was that for successful implementation, a diabetes prevention program should be community-owned. There was evidence of already existing readiness by the community to receive and facilitate the program. This aligns with previous reports that highlight the benefits of community-owned programs in remote Aboriginal settings [37,38,39]. Community-owned programs that embraced the authority and autonomy shared across community members were highlighted as successful and effective in the Australian setting [40,41,42,43]. A literature review of community intervention strategies that specifically aimed to improve and maintain the general health and well-being of the Aboriginal and Torres Strait Islander people indicated that those intervention programs where members of the community defined their strengths and gaps and owned solutions to their problems successfully fostered community empowerment and effective implementation of the program. The review also highlighted the need for an appropriate evaluation methodology to further strengthen the evidence for program effectiveness [44]. These findings align with the suggestions raised by the cultural advisors who participated in our study, who advocated for a thorough evaluation plan of the proposed diabetes prevention program. 

Our second finding highlighted the need for training and upskilling local facilitators from the community for program facilitation. This is closely related to the ownership facet of the program. Identifying community ‘brokers’ who understand the value of the proposed program and assigning facilitation to them is key in utilising the existing strength and capacity within the community [45]. Community facilitation has been reported to lead to successful program delivery through developing mutual trust between facilitator and participants, understanding priorities of the community and being flexible about program delivery [45]. Successful program delivery also relies on strong partnerships and collaborations [46]. Current study participants were potential facilitators for the program including health promotion officers and community engagement officers who expressed their desire to facilitate the proposed program. There was evidence of readiness from service providers who participated in the consultation to support the proposed program as it would fill an existing gap in the existing required services. Our study consultations further indicate that it is important to provide appropriate training regarding program sessions and facilitation, and to provide ongoing support from health care experts for the proposed program facilitators. This is well aligned with the original TTP program training components [21].

Another important finding from the study was the significance of culturally appropriate program delivery. Cultural appropriateness draws from when the worldviews of the community are prioritised in program design and delivery [47,48]. Previous work has highlighted the significance of prioritising First Nations worldviews and the importance of relationships and social connectedness as positive intrinsic determinants for obesity expression [49]. The Western biomedical model that emphasised calorie counts, diet restrictions, and exercise time were not valued as metrics of well-being among the Aboriginal participants of their study and were considered less effective in promoting lifestyle changes [49]. Several obesity prevention programs involving Aboriginal children have similarly identified the importance of incorporating careful and culturally appropriate facilitation approaches [25,26,27]. Involvement of young facilitators as well as Elders in the program was recommended in this study to promote effective program delivery. Youth participation is important to sustain changes as well as to equip the emerging generation with leadership skills [45]. Young facilitators have the potential to influence and inspire young participants in the program using a ‘big brother/sister’ approach as quoted by one of the participants during consultation. Participation of Elders in this program is equally critical in terms of mentoring the young champions as well as sharing their lived experiences with the families [50]. Sharing lived experiences has been reported to be an effective and preferred method of learning for many Aboriginal people [49,51], as also proposed in the current consultation by several Aboriginal community engagement officers who showed readiness to facilitate the proposed program. Elder’s participation also instigates intergenerational knowledge transfer [45].

Our findings also emphasize the importance of acknowledging the environmental and social context of the community while implementing the adapted TTP program. The complex interplay of wider social determinants, while instigating behaviour changes through lifestyle intervention programs (such as TTP), has been highlighted in the literature [52,53]. While exploring the interactions between individual agency and societal influences, it was recommended that addressing the core social and environmental elements of the society is crucial to influence lifestyle changes [52]. Through our consultations, a series of factors relating to food insecurity including higher cost for healthy food items, lack of access to fresh fruits and vegetables, as well as co-morbidities of parents or caregivers and cultural obligations were highlighted by the participants as important determinants of lifestyle changes. While implementing the adapted TTP program, it is crucial to consider the social and environmental factors for successful and sustainable outcomes.

A major strength of this study was that it was designed in response to a community need. The study design and the delivery were led by Aboriginal researchers (L.M., K.C.) in partnership with Congress. Other strengths include in-depth consultation with a range of sectors of the community and prioritising Aboriginal perspectives. The active involvement of the local Aboriginal research officer (S.M.) to ensure local Aboriginal interpretation of data was key. However, the study is limited to perceptions and opinions of the study participants in one Central Australian urban setting as well as from a limited number of family groups. Hence, the findings may not represent all family groups or cannot be generalizable to other communities in or beyond Central Australia.

## 5. Conclusions

Through actively engaging families, Elders and service providers, the current study provides recommendations for adapting a youth diabetes prevention program appropriate for the Central Australian context. This work highlights the identified need for such a program and recommends it is community owned and led. The approach taken to engage key stakeholder groups in the adaptation process in this study was important to meaningfully incorporate community perceptions. It is recommended that the proposed program is piloted in remote and urban community settings in Central Australia, led by a community-controlled health service.

## Figures and Tables

**Table 1:** Key characteristics of the original Tribal Turning Point program.

Ownership	Research Team Had Ownership of the TTP Program
Facilitators	The program was delivered by local people, referred to as ‘health coaches’, who were members of the tribe, or non-Natives integrated to the tribe.
Incentives	The TTP program offered incentives for participants to encourage attendance and meeting weekly behavioural goals.
Formative work	The mode of delivery and curriculum was informed by community consultations including First Nations American youth and parents. Engagement strategies, cultural appropriateness, resources required for the program were discussed through focus groups and workshops.
Program layout and delivery	Ten group sessions with three motivational individual family sessions over 4 months. An additional ‘booster program’ including two group sessions and two motivational interviews session were provided after the core program delivery over 4 months. The session frequency was weekly, and duration was approximately two hours per session.
Program location	The program was delivered usually at a community hall or easily accessible location determined by each state/community.
Program sessions	Designed for children and attended by the child and one parent or caregiver. Sessions included 45 min fun physical activity, food preparation, cultural activities, interactive learning, parent information (parenting, nutrition and health) and a group meal. The motivational sessions explored the health and behavioural goals discussed at group sessions, individualized for each family.
Program content	Group sessions covered key messages such as ideal serves of vegetables and fruits, restricted screening time and recommended time for physical activity. The motivational interview sessions explored the health as well as goals developed from the group sessions with each family separately as individual sessions. Booster/revision sessions were held to recap key messages delivered through the program as well as to provide tips strategies to sustain the healthy habits.

**Table 2:** Aboriginal and Torres Strait Islander quality appraisal tool (CREATE).

Questions	Yes/Partially/No	Justification
Did the research respond to a need or priority determined by the community?	Yes	The study is in response to a priority set by the Aboriginal and Torres Strait Islander Advisory Group and the Clinical Reference Group of the Diabetes across Lifecourse Northern Australia Partnership (Partnership).
Was community consultation and engagement appropriately inclusive?	Yes	The study integrated community perspectives to adapt a behavioural intervention program to prevent diabetes among Aboriginal children in Central Australia. Aboriginal family groups, a range of health service providers, school teachers, and cultural advisors from the community were engaged via workshops and focus groups for consultation. There was also ongoing engagement between the employed local community researcher (S.M.) and the community due to the connections and relationships of the researcher with the community.
Did the research have Aboriginal and Torres Strait Islander research leadership?	Yes	The study funding application was co-led by a senior Aboriginal researcher (L.M.). Data collection and data interpretation were co-led by Aboriginal researchers L.M. and S.M. Aboriginal researchers S.T. and K.C. were members of the working groups that oversaw community consultations and resource development process for the adapted intervention program.
Did the research have Aboriginal and Torres Strait Islander governance?	Yes	The study is nested within the governance of the Diabetes across Lifecourse Northern Australia Partnership, a partnership between researchers, policy makers and health services, including Aboriginal community-controlled health services. The Partnership’s Aboriginal and Torres Strait Islander Advisory Group (represented by S.M.) advised on research priorities, community engagement and ensures that research is conducted in a culturally safe manner. In addition, a cultural reference group was formed to oversee the cultural aspects and language used in developing the adapted program manual. Terms of Reference for the cultural reference group were developed where regular meeting times and duration were agreed by all members.
Were local community protocols respected and followed?	Yes	Local community protocols were respected and followed under the guidance of the cultural reference groups led by local community health researchers (L.M., S.M.) and cultural advisors (S.T.).
Did the researchers negotiate agreements in regard to rights of access to Aboriginal and Torres Strait Islander peoples’ existing intellectual and cultural property?	Yes	The rights of access to Aboriginal and Torres Strait Islander peoples’ existing intellectual and cultural property are emphasized in each of the community consultation and engagement sessions, and also individually with participants through the informed consent process.
Did the researchers negotiate agreements to protect Aboriginal and Torres Strait Islander peoples’ ownership of intellectual and cultural property created through the research?	Yes	As stated in the study ethics document, to protect Aboriginal and Torres Strait Islander peoples’ ownership of intellectual and cultural property, the Aboriginal and Torres Strait Islander Researchers and the Partnership’s Aboriginal and Torres Strait Islander Advisory Group are consulted on what information is and is not appropriate for publication. All data are de-identified to protect all participants. The Aboriginal and Torres Strait Islander Advisory Group and the working groups specifically formed for the current project will guide appropriate processes in the event any unforeseeable event in relation to intellectual property occurs. Cultural property shared by the families will remain that of each individual family and if used to inform the study outcome, permission will be sought for inclusion.
Did Aboriginal and Torres Strait Islander peoples and communities have control over the collection and management of research materials?	Yes	The study was designed in a systematic manner (refer to Methods section) where timely progress of the adapted program was fed back to Aboriginal families and community members for their ongoing input on the adapted program’s design and resource development. We are committed to ensuring full understanding of the research by participants and communities through employment of a local Aboriginal community member to discuss recruitment and the consent process with each individual participant.
Was the research guided by an Indigenous research paradigm?	Yes	As stated under the ‘data analysis’ section, an Aboriginal cultural lens was adopted by prioritizing Aboriginal voices throughout the analysis and interpretation process by all researchers. All key Aboriginal and Torres Strait Islander stakeholders and Aboriginal and Torres Strait Islander researchers have been included as co-authors in publications. This has enabled Aboriginal values and perspectives to contribute to interpretation of the study findings.
Does the research take a strengths-based approach, acknowledging and moving beyond practices that have harmed Aboriginal and Torres Strait peoples in the past?	Yes	The current study acknowledges the gap in previous and existing obesity prevention programs for Aboriginal children. The current study employs a strengths-based approach to establish genuine partnerships with participating communities and to accommodate Aboriginal world views and suitable evaluation frameworks prior to implementing the prevention program.
Did the researchers plan and translate the findings into sustainable changes in policy and/or practice?	Yes—planned Partially for translation	The researchers planned translation from the inception of this study. This included partnership with organizations who are implementation and/or translation partners in this work—Congress, NT Health and NT Educations departments. The current study is informing the adaptation of a behavioural intervention program. A trial of the adapted program has the potential to translate the findings into policy or practice changes. The researchers have submitted applications to fund the trial of assessment of effectiveness and implementation of the adapted program.
Did the research benefit the participants and Aboriginal and Torres Strait Islander communities?	Yes	The project aims to improve the well-being of Aboriginal and Torres Strait Islander peoples by reducing risk of diabetes andheart disease in children who are at high risk for these chronic conditions. Another key benefit of the project is in harnessing multi-industry expertise in building and maintaining collaborative relationships for delivery and sustainability of the project beyond the research.

**Table 3:** Description of individuals who participated in program consultation.

Role/Occupation	Number	Aboriginal or Torres Strait Islander People	Females
Health promotion officers	3	2	3
Community engagement officers	2	2	1
Paediatricians	4	1	2
Child health nurses	4	0	4
Dietitians	6	0	5
Diabetes educator	1	1	1
Podiatrist	1	0	1
Physical education teacher	1	0	1
Teaching and learning officer	1	0	1
Cultural advisors	2	2	2
Elder	1	1	1
Parents/caregivers of children aged 5–10 years	12	12	6
Total	38	21	28

## Data Availability

The data presented in this study are available on request from the corresponding author. The data are not publicly available due to privacy reasons.
